# Molecular Aspects of Renal Immunology: Current Status and Future Perspectives

**DOI:** 10.3390/ijms23074040

**Published:** 2022-04-06

**Authors:** Theodoros Eleftheriadis, Marta Crespo

**Affiliations:** 1 Department of Nephrology, Faculty of Medicine, University of Thessaly, 41110 Larissa, Greece; 2 Nephrology Department, Institut Hospital del Mar d’Investigacions Mèdiques, Hospital del Mar, Parc de Salut Mar, 08003 Barcelona, Spain; mcrespo@parcdesalutmar.cat

Kidney transplantation is the most promising treatment available for patients with end-stage kidney disease. However, despite the application of immunosuppressive protocols, long-term graft survival remains relatively short, mainly as a result of immunological issues [1]. The Special Issue, “Molecular aspects of renal immunology: Current status and future perspectives”, examines and analyzes factors affecting the interaction between the host immune system and the kidney transplant that result in graft failure, giving particular attention to new methods for the early detection of graft rejection.

Very early events, starting from the kidney graft harvesting, may affect the recipient’s immune response and eventually impact long-term graft survival. Unfortunately, the shortage of available kidney transplants has resulted in the use of non-optimal grafts, which are more susceptible to ischemia–reperfusion injury (IRI)-induced acute tubular necrosis, resulting in the increased frequency of delayed graft function [2]. Delayed graft function is associated with a higher incidence of acute rejection [3,4]. In this Special Issue, Fernandez and al. reviewed the effect of IRI on kidney graft survival [5]. The metabolic stress and the subsequent reactive oxygen species overproduction occurring during kidney transplant ischemia–reperfusion induces cell apoptosis and necrosis, and the release of damage-associated molecular patterns. The latter are recognized by specific receptors, such as Toll-like receptors, leading to innate immune cell activation and the production of several cytokines and chemokines. This inflammatory environment induces further renal cell necrosis and recruits adaptive immune system cells that facilitate graft rejection. As noted in this review [5], new therapeutic strategies are currently under development and clinical evaluation to attenuate IRI. These include advances in kidney graft preservation with novel perfusion machines, as well as pharmaceutical or biological interventions. Ferroptosis inhibitors, the regulation of complement cascade, and the manipulation of regulatory cells may play a role in reducing IRI after transplantation. In our opinion, the emerging organ-preservation technologies will allow graft reconditioning and manipulation, with the help of medications and gene silencing methods that aim to ameliorate IRI and the subsequent activation of the immune system.

Besides the release of damage-associated molecular patterns that activate the recipient’s immune system, IRI also affects the renal endothelium glycocalyx. The glycocalyx consists of proteoglycans, glycoproteins, and glycolipids [6]; it is a significant part of the glomerular filtration barrier and plays a role in the pathogenesis of various kidney diseases such as diabetic nephropathy [6,7]. In this Special Issue, Duni et al. reviewed the effect of IRI on kidney transplant endothelium glycocalyx, and its consequences on the host immune response [8]. Disintegration of the endothelial glycocalyx induced by IRI exposes the denuded endothelial cells of the graft to further inflammatory and oxidative damage. There are complex links between the shedding of glycocalyx components—such as syndecan-1, hyaluronan, heparan sulfate, and CD44—and the activation of immune system components including Toll-like receptors, proinflammatory transcription factors, and cytokines. Currently, experimental strategies are being developed for protecting the endothelial glycocalyx, and promising novel nephroprotective molecules such as sphingosine-1 phosphate have been revealed. Since modern technology can facilitate the high throughput visualization and analysis of the endothelial glycocalyx, it may be appropriate for clinical studies to move towards reducing graft glycocalyx IRI and the subsequent immune response.

As noted, perioperative kidney transplant IRI may enhance the recipient’s immune response, consequently resulting in a shorter graft survival time. However, the kidney graft stimulates the immune system of the host continuously. At present, three T cell allorecognition pathways have been identified [9]. In the direct T cell allorecognition pathway [9], recipient CD8+ and CD4+ T cells recognize intact major histocompatibility complex (MHC) Class I and MHC Class II molecules, respectively, on donor professional antigen-presenting cells (APCs) that are transferred with the graft. Since 10% of recipient T cells recognize a single MHC alloantigen, the direct pathway is the most potent immune response against the graft. At present, because the recipient immune system eliminates donor-derived APCs shortly after transplantation, it is believed that the direct pathway plays a role during the immediate post-transplantation period [9]. In the indirect T cell allorecognition pathway, graft antigens—mostly MHC molecules—are internalized, processed, and presented by recipient APCs to recipient T cells. The indirect pathway can become active at any time, and is responsible for late graft cellular and humoral rejection [9]. Finally, in the semi-direct T cell allorecognition pathway, graft MHC molecules are acquired by recipient dendritic cells and presented intact to recipient T cells. However, the exact role of the semi-direct pathway remains to be elucidated [9].

This Special Issue includes an experimental study by Eleftheriadis et al. [10] which challenges the above generally accepted paradigm in cultures of primary human renal proximal tubular epithelial cells (RPTECs). In the study, all the required molecules for CD4+ T cell activation (HLA-DR, CD80, and ICAM-1) were detected RPTECs. The co-cultures of RPTECs with alloreactive CD4+ T cells activated the T cell receptor and co-stimulation signal transduction pathways, and induced T cell proliferation. Interestingly, anoxia-reoxygenation decreased HLA-DR and CD80 expression, but increased ICAM-1. However, due to ICAM-1 overexpression, the antigenicity of RPTECs increased, since anoxia-reoxygenation-treated RPTEC co-cultures with alloreactive CD4+ T cells resulted in the increased activation of the T cell receptor and co-stimulation pathways, as well as T cell proliferation. FOXP3 remained unaffected, indicating that proliferating T cells were not differentiated towards a regulatory phenotype. These results signify that kidney graft antigenicity remains high, even after the first post-transplantation period, since RPTECs are subject to direct allorecognition. Moreover, it has been indicated that, at the immediate post-transplantation period, IRI may enhance the antigenicity of the kidney graft. Hence, efforts to alleviate kidney transplant IRI by evolving and applying more efficient preservation methods may prove beneficial in treatment. Finally, since ischemia–reperfusion increases RPTECs’ antigenicity by enhancing ICAM-1 expression, the possible therapeutic effect of blocking the interaction between cell adhesion molecules deserves evaluation.

Despite the progress in elucidating the mechanisms involved in kidney graft dysfunction by the host immune response, a huge amount of ground still has to be covered to achieve a more accurate and early detection of graft rejection in the clinic. Kidney biopsy is the gold standard for assessing the interaction between the recipient’s immune system and the kidney graft. It discriminates the various types of rejection and determines their severity. Moreover, surveillance kidney biopsies allow the identification of subclinical rejection and early treatment. However, despite advances in the histopathology of kidney transplant classification, reflected by the many revisions of the generally accepted Banff calcification system, there are borderline cases of graft rejection in which clinical decision is difficult [11]. Usually, the detection of borderline changes in biopsies for cause leads to anti-rejection treatment; however, in many centers, patients with the same diagnosis in surveillance biopsies are not treated. Another common finding in kidney transplant biopsies is interstitial fibrosis and tubular atrophy (IFTA). Its presence in biopsies can be associated with T cell-mediated rejection, antibody-mediated rejection, and recurrent glomerulonephritis, among other diseases. Sometimes IFTA cannot be associated with any post-transplant disease [12]. Hence, the molecular evaluation of kidney biopsy specimens may lead to more accurate diagnoses, and avoid sub- or over-exposure to immunosuppressives.

In this Special Issue, Chamoun et al. evaluated rejection-related gene expression in subclinical rejection and biopsies with borderline changes or IFTA. A rejection-associated gene (RAG) score containing 109 genes derived from normal and clinical rejection was employed to classify the study groups. A positive RAG score was observed in 83%, 38%, 17%, 25%, and 5% of subclinical rejections, borderline changes in biopsies for cause, borderline changes in surveillance biopsies, IFTA in biopsies for cause, and IFTA in surveillance biopsies, respectively. Considering outcome as death-censored graft loss, or a glomerular filtration rate decline greater than 30% at 2 years, a positive RAG score was an independent predictor of graft outcome from histological diagnosis (hazard ratio: 3.5). Thus, a positive RAG score can predict graft outcome in surveillance and for cause biopsies with a less severe phenotype than clinical rejection.

In conclusion, the articles of the present Special Issue indicate that, with the assistance of constantly evolving modern technology and medicine, there are great opportunities for prolonging kidney graft time and patient survival. In the near future, measures for ameliorating IRI at the perioperative period, better understanding the alloimmune response, and the use of advanced technology for the early and accurate diagnosis of graft rejection are expected to extend graft survival.
